# ZmHOX32 is related to photosynthesis and likely functions in plant architecture of maize

**DOI:** 10.3389/fpls.2023.1119678

**Published:** 2023-03-22

**Authors:** Xinxin Miao, Wanchao Zhu, Qixiao Jin, Zemeng Song, Lin Li

**Affiliations:** ^1^ National Key Laboratory of Crop Genetic Improvement, Huazhong Agricultural University, Wuhan, China; ^2^ Hongshan Laboratory, Wuhan, China

**Keywords:** maize, ZmHOX32, leaf, photosynthesis, tsCUT&Tag

## Abstract

HOX32, a member of the HD-ZIP III family, functions in the leaf morphogenesis and plant photosynthesis. However, the regulatory mechanism of HOX32 in maize has not been studied and the regulatory relationship in photosynthesis is unclear. We conducted a comprehensive study, including phylogenetic analysis, expression profiling at both transcriptome and translatome levels, subcellular localization, tsCUT&Tag, co-expression analysis, and association analysis with agronomic traits on HOX32 for the dissection of the functional roles of HOX32. *ZmHOX32* shows conservation in plants. As expected, maize HOX32 protein is specifically expressed in the nucleus. *ZmHOX32* showed constitutively expression at both transcriptome and translatome levels. We uncovered the downstream target genes of ZmHOX32 by tsCUT&Tag and constructed a cascaded regulatory network combining the co-expression networks. Both direct and indirect targets of ZmHOX32 showed significant gene ontology enrichment in terms of photosynthesis in maize. The association study suggested that *ZmHOX32* plays an important role in regulation of plant architecture. Our results illustrate a complex regulatory network of HOX32 involving in photosynthesis and plant architecture, which deepens our understanding of the phenotypic variation in plants.

## Introduction

It is predicted that the world population will reach up to 9 billion in 2050. According to the current food production, only 70-90% of human can be fed, which largely lags behind our needs for food in future. Increasing food production is the only option (Salas Fernandez et al., 2009).

Maize (*Zea mays* L.), a most widely planted crop in the world, is particularly important in ensuring food security (Assefa et al., 2018). Photosynthesis enables the synthesis of carbohydrates, which plays an important role in guaranteeing yield of crops as well as maize. Similar to other C4 plants, the carbon reaction of maize is also carried out in mesophyll cells and vascular bundle sheath cells (Dai et al., 2022). The C4 photosynthetic pathway is very complicated, where numerous genes are involved in the regulation. However, the regulatory relationships of photosynthesis related genes are still limited.

Leaf is the main place of photosynthesis, respiration, and transpiration in plants. Plant architecture traits such as leaf size, shape, thickness, and angle can affect the photosynthesis rate through regulating the utilization rate of light energy, thereby affecting the accumulation of carbohydrates. Therefore, the improvement of plant architecture is also one of the main goals of crops breeding. Plant leaves start from the flanks of the stem apical meristem, begin to develop asymmetrically, and establish three-dimensional spatial polarity along three directions: the base-apex axis, the middle-edge axis, and the adaxial-distal axis. Among them, the paraxial maintenance-distal axial patterning plays a crucial role in leaf morphogenesis, which is caused by antagonism between specialized adaxial and abaxial tissue-specific genes (Moon and Hake, 2011).

Many studies have reported that HD-ZIP III is involved in the regulation of leaf morphogenesis. HD-ZIP protein genes (homeodomain leucine zipper), a class of plant-specific transcription factors, belong to the homeobox family because of the containment of highly conserved homeodomain (HD) and leucine zipper structures domain (leucine zipper, LZ). Besides the basic HD and LZ domains, HD-ZIP III family proteins also contain a START domain that is able to bind to steroid ligands, which is highly conserved in evolution (Ponting and Aravind, 1999; Schrick et al., 2004). Moreover, several HD-ZIP III family proteins also contain a MEKHLA (Met-Glu-Lys-His-Leu-Ala) domain that consists of 6 conserved amino acids. This domain is involved in the signal transduction pathways mediated by chemical and physical stimulation, and play a potential role in affecting plant photosynthesis (Mukherjee and Bürglin, 2006). *Hox32* is a member of HD-ZIP III family genes. In rice, overexpression of *OsHOX32* caused narrow adaxial curling leaves, reduced leaf angle, erect plant type, dwarf plants, and reduced chlorophyll levels, thus repressing the photosynthesis efficiency (Li et al., 2016). In *Arabidopsis*, *Hox32* homologs *IFL1*, *ATHB-9* and *ATHB-14* are involved in the development of the apical meristem, vascular bundle and the paraxial region of the lateral tissue, regulating the embryonic development process in the formation of the root tip (Palena et al., 2001). However, this gene has not been studied in maize at present and the regulatory network of *Hox32* in maize is largely unknown.

To the end, we explore the function of *ZmHOX32* using a cutting-edge molecular technique tsCUT&Tag and dissect the functional roles of *ZmHOX32* in plant architecture by association mapping on a global diverse association panel. The cascading regulatory network in photosynthesis and association signals with plant architecture of *ZmHOX32* may lay a foundation for the improvement of plant architecture and photosynthesis in maize.

## Experimental process

### Bioinformatics analysis for *ZmHOX32*

The *cis*-acting elements of the *ZmHOX32* gene promoter were predicted using Plantcare (Rombauts et al., 1999)^1^. The phylogenetic tree of *ZmHXO32* and other plant homologous proteins was constructed using the NJ method in MEGA software (Kumar et al., 1994). The conserved structural domains of *ZmHOX32* and other homologous proteins were detected using the NCBI Conserved Domain Database (CDD) search tool (Marchler-Bauer, 2002)^2^.

### Vector construction

The pM999-GFP vector was digested using Xba1 under condition 37 °C for 2-3 h. The CDS of the *ZmHOX32* with removement of stop codon was inserted into the pM999-GFP vector upstream the GFP sequence, the amplification primers are shown in **Supplementary Table 1**. PCR fragment and linearized vector were recombined using ClonExpress^®^ II One Step Cloning Kit (Vazyme C113-02), and the resultant was transformed into DH5α. The transformed bacterial solution was evenly coated on LB medium plates that contain Ampicillin and incubating for 12 h-16 h at 37 °C. Positive colonies were filtered by PCR and sanger sequencing. After expanding the cultivation of positive clone, the plasmid was extracted using the Endo-Free Plasmid DNA Maxi Kit (OMEGA D6926-03).

### Isolation and transformation of protoplasts

Protoplasts were isolated from yellowing seedlings grown in dark culture for about 9-11 days at the nutritional growth V3 stage. The plasmids were transformed into protoplasts according to the described method (Yoo et al., 2007). The GFP fluorescence signal was observed under a confocal microscope (Leica) with a 485 nm laser. The protoplasts with successful transformation were subsequently subjected to subcellular localization and CUT&Tag.

### tsCUT&Tag experimental procedure

A new cutting-edge technique tsCUT&Tag was employed for the dissection of regulatory network of *ZmHOX32* (Wu et al., 2022). The Hyperactive *In-Situ* ChIP Library Prep Kit for Illumina (pG-Tn5) kit (Vazyme TD901) was used for the operation. Transformed protoplasts were observed by fluorescence microscopy to measure transformation efficiency. The samples with transformation efficiency no less than 60% were selected for subsequent CUT&Tag experiments. Two biological replicates were set for the constructed vector containing CDS of *ZmHOX32*. Cells were collected by low-speed centrifugation at 100 r/min for 2 min, and resuspended using 100 μl resuspension solution. After treating the resultant with ConA beads, incubation was performed with GFP antibody and corresponding secondary antibody. pG-Tn5 Transposon was used to fragment the DNAs and insert adaptors. Finally, the fragmentated DNA was extracted for library construction. After quantifying by Qubit, the constructed libraries were sequenced with pair-end 150 bp in Illumina Hiseq X-Ten platform. The transformed protoplasts with pM999-GFP vector were as the control group.

### Data analysis for tsCUT&Tag

The reads were mapped into B73 reference genome (AGPv4) using Bowtie2 (Langmead and Salzberg, 2012) with the parameters “-p 10 –phred33 -I 0 -X 1000 –no-discordant –no-mixed”. PCR duplicates and reads with low quality (mapping quality score < 30) were removed. The searching for high confidence peaks (peaks p < 1×10^-5^) was performed using MACS (Feng et al., 2012) with the parameters “callpeak -g 2.2e+9 -s 150 -B -p 1e-5 -f BAMPE”. The distribution of peaks over the whole genome was analyzed using the ChIPseeker (Yu et al., 2015) in R. If the peak is located within the range of 3 kb upstream to 3 kb downstream of the gene, we will assume that this gene is the target of the protein. The performance was finished by the intersect function of the BEDtools software (Quinlan and Hall, 2010). GO enrichment analysis of target genes was performed using the AgriGOv2 (Tian et al., 2017)^3^ and the enriched GO terms were visualized using R. The generated downstream target genes were presented in **Supplementary Table 2**.

### Association analysis

An association analysis was performed using genotypes and phenotypes of 690 inbred lines of maize with the general linear model (Abe et al., 2012; Gui et al., 2020). The phenotypes contained Tassel main axis length, Tassel branch number, Silking time, Pollen shed, Plant height, Leaf number above ear, Kerner number per row, Kernel width, Kernel thickness, Kernel length, Heading date, Ear length, Ear leaf width, Ear leaf length, Ear height, Ear diameter, Cob weight, Cob diameter, 100 Grain weight. All 19 phenotypes were distinguished by two categories of plant architecture and yield. Among them, Ear length, ear diameter, and cob diameter are contained in both of two types. The two types of phenotypic data were dimensionally reduced using R function “prcomp” and the setting “retx=T,scale=T,center=T”. The first two PCs were corrected for normal distribution by the R function “qnorm”. After the above processing, we obtained four phenotype data: Yield-pc1, Yield-pc2, Plant architecture-pc1, Plant architecture-pc2. In order to retain high-confidence SNPs, a total of 4069278 SNPs were retained through the filtering of VCFtools. (–maf 0.05 –minDP 5 –remove-indels –max-missing 0.9 –min-alleles 2 –max-alleles 2) (Danecek et al., 2011). Tassel software was used for SNP sorting and hapmap format conversion (Bradbury et al., 2007). Finally, the processed hapmap files and phenotype data were used as input for GWAS analysis by GAPIT software (Lipka et al., 2012). The threshold for significant SNPs is -Log_10_(1/4069278), which is 6.609517.

## Results

### Phylogenetic tree of *ZmHOX32* demonstrates the potential functional conservation of HOX32 proteins across different plants

**Figure 1:**
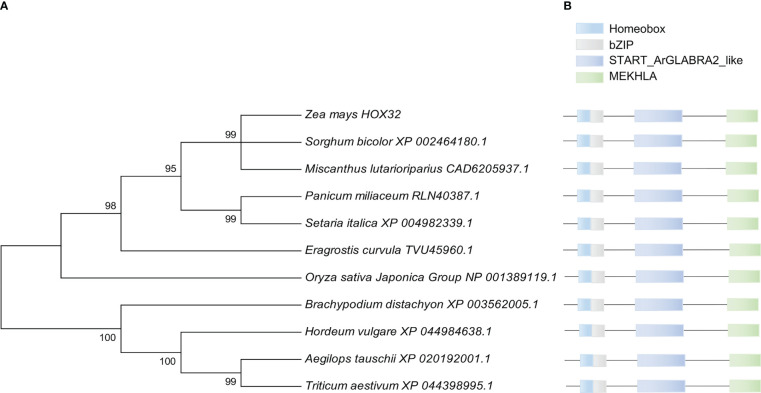
Phylogenetic tree and conserved domain analysis of *ZmHOX32* with different plants. **(A)** Construction of phylogenetic tree using MEGA7. **(B)** Conserved domains of HOX32 proteins in different plants.

HD-ZIP III gene family is one of the important transcription factor family. Rice *OsHOX32* has been reported to function in leaf morphogenesis. Comparative genomics has identified that *Zm00001d033246* in maize is homologous to *OsHOX32* in rice and so named as *ZmHOX32*. *ZmHOX32* contains 18 exons, with 3,259 bp in genomic length and 2,570 bp of CDS, which encodes a HOX protein of 856 amino acids. To sense the potential function of *ZmHOX32*, we constructed a phylogenetic tree of HOX32 in plants and found that the closest evolutionary homologs are *XP_002464180.1* in *Sorghum bicolor* and *CAD6205937.1* in *Miscanthus lutarioriparius*, both of which are C4 crops. Further analysis of the conservation of HOX32 proteins showed that HOX32 is conserved across different plants with nearly identical functional domains, suggestive that HOX32 genes are likely to have conserved function in plants (**Figure 1**).

### Expression pattern of *ZmHOX32*

**Figure 2:**
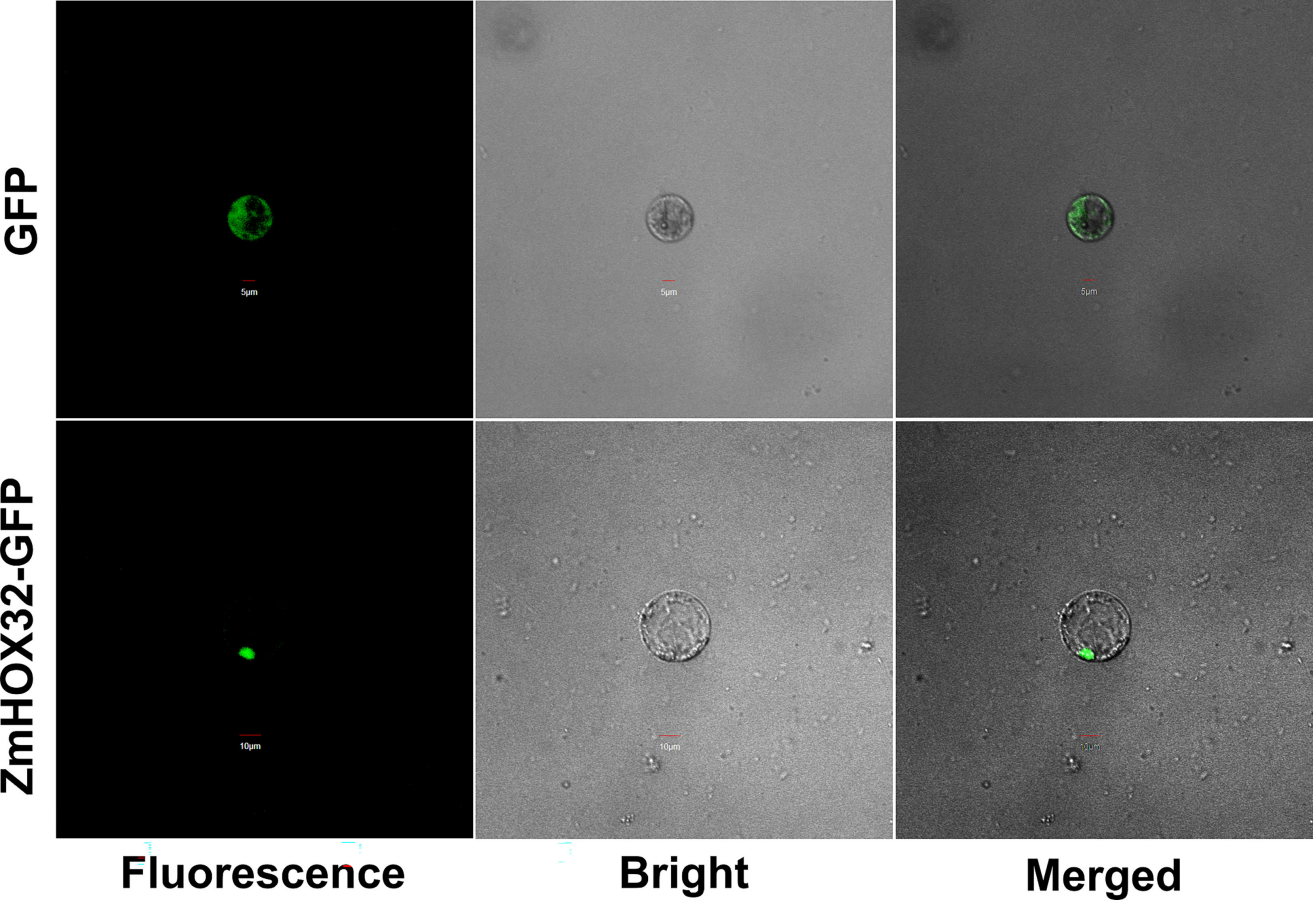
Subcellular localization of the *HOX32* protein in maize. Subcellular localization of 35S::GFP and 35S::ZmHOX32-GFP in maize protoplasts; maize protoplast cells were used for taking images of green fluorescence, chloroplast autofluorescence, visible light, and merged visible light.

To clarify the location of HOX32 protein in cells, we constructed a vector with the coding sequence of *ZmHOX32* that fused to the upstream of green fluorescent protein (GFP) sequence and performed a subcellular localization assay. Successfully fused plasmid and empty vector were delivered into maize protoplasts to generate transient expression by infiltration. The GFP signal in empty vector (as control) was detected throughout the whole cell. In contrary, the ZmHOX32-GFP signal is specifically detected in the nucleus of maize protoplast cells (**Figure 2**). These results suggested that ZmHOX32 protein tends to function in nucleus, which is compatible with the intrinsic functional role as a transcription factor.

**Figure 3:**
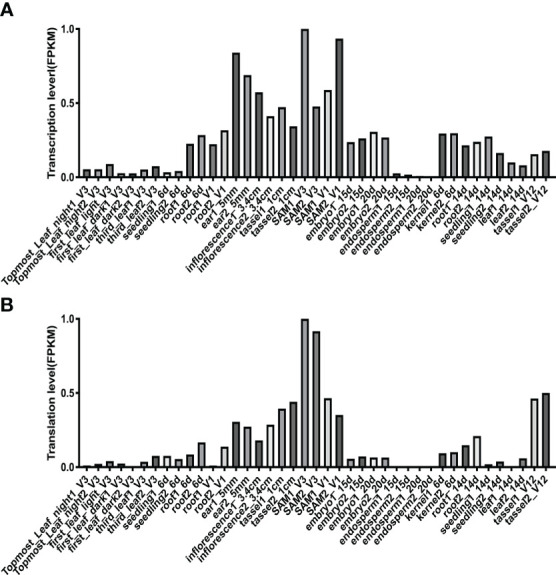
Expression of *ZmHOX32* gene in maize tissues. **(A)** Transcription of *ZmHOX32* gene. **(B)** Translation of the *ZmHOX32* gene.

To profile the expression pattern of *ZmHOX32* across maize development, we extracted a comprehensive transcriptome and translatome data from 33 different tissues or stages of maize different development from a previous study (Han et al., 2023). The expression abundance of *ZmHOX32* was detected in roots, stems, leaves, and other tissues (**Figures 3A, B**). Interestingly, we noticed that *ZmHOX32* was preferentially expressed in SAM and tassel etc, suggesting a potential function in plant development.

### Potential upstream regulators of *ZmHOX32* are associated to light responsive

To identify and characterize the upstream regulators of *ZmHOX32*, the PlantCARE (Rombauts et al., 1999) database was used to analyze the promoter sequence of *ZmHOX32*, which suggested that *ZmHOX32* gene contains a variety of functional response elements (**Table 1**). Besides the TATA-box and CAAT-box that belonged to the basic core element of the promoter, there were several specific motifs with potential different functions through the binding of the upstream regulators. It has been reported that TCA-element is involved in salicylic acid signal transduction pathway and GC-motif plays an important role in antioxidant response (Zhou et al., 2019). Some motifs are associated with hormone signal or response, such as CGTCA-motif, TGA-element, ABRE and P-box. Furthermore, we identified six different motifs that all related to light responsive. These *cis*-acting elements include Box 4, AE-box, G-Box, GT1-motif, ACE and Sp1. These results suggested that *ZmHOX32* was likely to be regulated by different genes that associated to photosynthesis.

**Table 1:** *ZmHOX32* gene promoter *cis*-acting elements.

Name of cis element	Source plant	Site	Signal sequence	Function
TCA-element	*Nicotiana tabacum*	1392	CCATCTTTTT	salicylic acid responsive element
GC-motif	*Zea mays*	1123	CCCCCG	anoxic specific inducibility responsive element
CGTCA-motif	*Hordeum vulgare*	832	CGTCA	MeJA responsive element
CCAAT-box	*Hordeum vulgare*	470	CAACGG	MYBHv1 binding site
TGA-element	*Brassica oleracea*	264	AACGAC	auxin responsive element
ABRE	*Arabidopsis thaliana*	1497	AACCCGG	abscisic acid responsive element
CAT-box	*Arabidopsis thaliana*	1748	GCCACT	regulation of plant meristem expression
P-box	*Oryza sativa*	865	CCTTTTG	gibberellin responsive element
Box 4	*Petroselinum crispum*	145	ATTAAT	light responsive element
AE-box	*Arabidopsis thaliana*	405	AGAAACTT	light responsive element
G-Box	*Zea mays*	828	CACGTG	light responsive element
GT1-motif	*Arabidopsis thaliana*	907	GGTTAA	light responsive element
ACE	*Petroselinum crispum*	1367	GACACGTATG	light responsive element
Sp1	*Oryza sativa*	1861	GGGCGG	light responsive element

### Interaction proteins of ZmHOX32 are associated with photosynthesis

**Figure 4:**
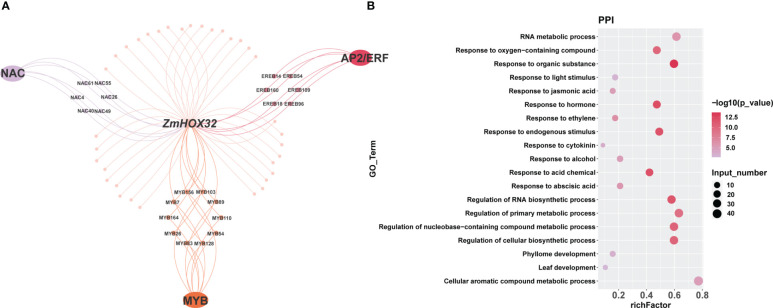
Enrichment of intercalating proteins of ZmHOX32. **(A)** Regulatory network of the intercalating proteins of ZmHOX32. **(B)** Degree of enrichment of intercalating proteins of ZmHOX32.

To explore the interaction proteins of ZmHOX32, we analyzed the data generated by RLL-Y2H-seq in maize (Yang et al., 2018; Han et al., 2023). We found that ZmHOX32 was likely to interacted to the proteins encoded by the genes of NAC, AP2/ERF, and MYB families (**Figure 4A**). GO enrichment analysis of these genes showed that they are correlated to hormone signaling, leaf development, and response to light stimulation (**Figure 4B**). These results implied complicated function of ZmHOX32, which may be related to photosynthesis.

### A regulatory network showed the complicated function of *ZmHOX32* in participating photosynthesis

**Figure 5:**
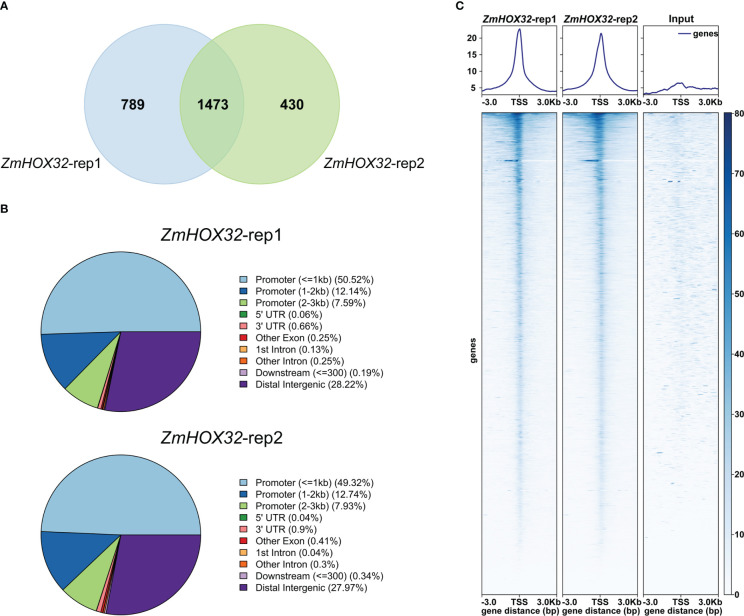
tsCUT&Tag reveals the genome-wide binding position of *ZmHOX32*. **(A)** The overlap between the downstream target genes of the two replicates of *ZmHOX32*. **(B)** Genome-wide binding site map of *ZmHOX32* two repeats. **(C)** Distribution of *ZmHOX32* tsCUT&Tag peaks at 3 Kb upstream and downstream of the gene.

To uncover the downstream target genes of *ZmHOX32*, we performed a tsCUT&Tag assay to investigate the binding sites of ZmHOX32 protein in maize B73 genome. The library and sequences showed good quality. The library fragments ranged from 200 to 650 bp, without primer dimer contamination (**Supplementary Figure 1A**). Sequencing results showed that the quality values of most bases were above 30, suggesting the good quality of this data (**Supplementary Figure 1B**). We also performed correlation analyses for the two replicates, which showed high correlations (Pearson Correlation Coefficient = 1, Spearman Correlation Coefficient = 0.98) (**Supplementary Figures 1C, D**). These results indicate the effective of tsCUT&Tag experiment. Two biological tsCUT&Tag replicates detected 2,262 and 1,903 target genes, respectively. Of these targets, 1,473 target genes were detected by both replicates and considered to be high-confidence targets (**Figure 5A**). Transcription factors usually bind to *cis*-acting elements in promoter of targets to determine the transcription of downstream genes (Lambert et al., 2018). So, we scanned the binding sites of *ZmHOX32* across the whole genome and uncovered that they were mainly located in promoter 3kb regions of target genes, which accounted for 70.25% and 69.99% of all binding sites in two replicates (**Figure 5B**). The signal heat map confirmed that the reads of tsCUT&Tag were significantly enriched near the transcription start site (TSS) (**Figure 5C**). These results demonstrate a genome-wide binding landscape of *ZmHOX32*, which provides us an unprecedented resource to dissect the function of *ZmHOX32* in maize.

**Figure 6:**
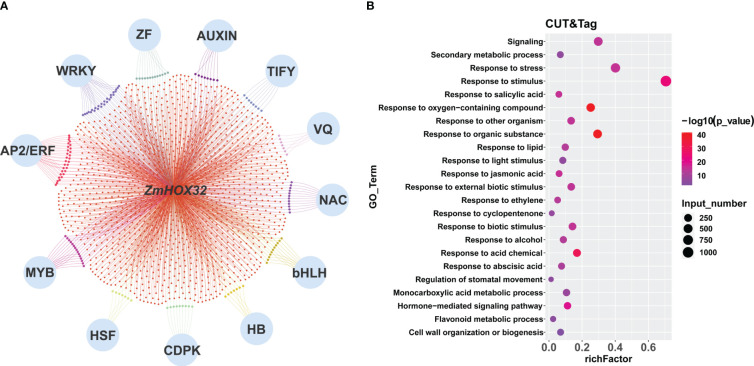
Enrichment of downstream target genes of *ZmHOX32* protein. **(A)** Regulatory network of downstream target genes of HOX32 protein. **(B)** Go enrichment for downstream target genes of HOX32 protein.

The target genes of *ZmHOX32* were significantly enriched in multiple gene families, such as WRKY, AUXIN, AP2/ERF, MYB and so on (**Figure 6A**), which are mainly related to plant growth, development and signal transduction, as well as photosynthesis in different plants (Chuck et al., 2008; De Boer et al., 2011; Zheng et al., 2018; Dickinson et al., 2020; Feng et al., 2020; Zhang et al., 2021). GO enrichment for these genes also revealed that most of them were involved in hormone signal transduction, development, and response to light stimulation (**Figure 6B**). These results are in line with the expectation and suggest the complicated functions of *ZmHOX32*.

**Figure 7:**
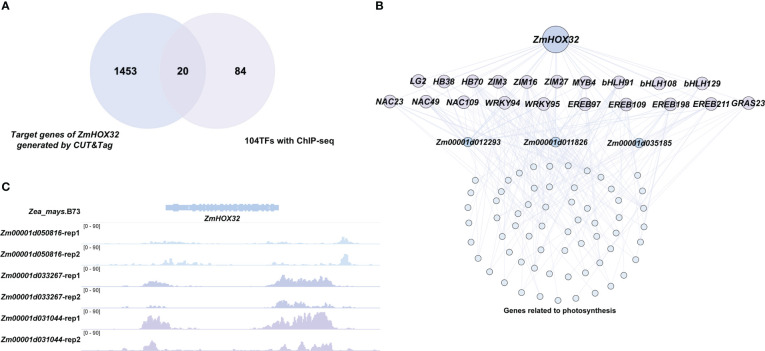
Characterization of the regulatory network of *ZmHOX32* using the ChIP-seq data of 104 transcription factors. **(A)** Overlap of *ZmHOX32* downstream target genes with 104 transcription factors. **(B)** Preliminary construction of the regulatory network of *ZmHOX32*. **(C)** Transcriptional binding sites of three *ZmHOX32* upstream transcription factors in the promoter region of *ZmHOX32*.

In previous study, the ChIP-seq of 104 TFs were performed in maize protoplasts to explore their potential target genes (Tu et al., 2020). These TFs are specifically expressed in leaf tissues, implying their possible role in light harvesting. Interestingly, we found 20 TFs of these 104 TFs could be targeted by HOX32 protein (**Figure 7A**), which is significantly higher than random (*P* value =8.13e-4). All the TFs belonged to the classical families associated with metabolism, signaling, transport, hormone, and cell wall (Tu et al., 2020) (**Supplementary Figure 2**). Of the targets of these 20 downstream TFs of ZmHOX32, 66 genes are annotated as photosynthesis-related genes that participated in light reaction (Thimm et al., 2004) (**Figure 7B**; **Supplementary Table 3**), suggesting a potential function of *ZmHOX32* in photosynthesis. In addition, we found three of the 66 photosynthesis-related genes were also directly targeted by the HOX32 protein. These three genes *Zm00001d012293* (FD4), *Zm00001d011826* (NDHO1) and *Zm00001d035185* (CDB1) have been evidenced to play important roles in photosynthesis (Hanke et al., 2005; Chen et al., 2022; Su et al., 2022). Furthermore, three leaf-related transcription factors - *Zm00001d050816* (ALF7), *Zm00001d033267* (bHLH43), and *Zm00001d031044* (bHLH163) were evidenced to directly target to *ZmHOX32* in a previous study (Tu et al., 2020) (**Figure 7C**). It has been shown that the transcription factors bHLH43 and bHLH163 play important roles in the light regulation mechanism of carotenoids and tricarboxylic acid cycle by light, respectively (Eprintsev et al., 2022; Xiang et al., 2022). These results demonstrate a regulatory module of *ZmHOX32* that affect photosynthesis in maize.

### Association analysis suggest *ZmHOX32* is also likely to function in plant architecture of maize

**Figure 8:**
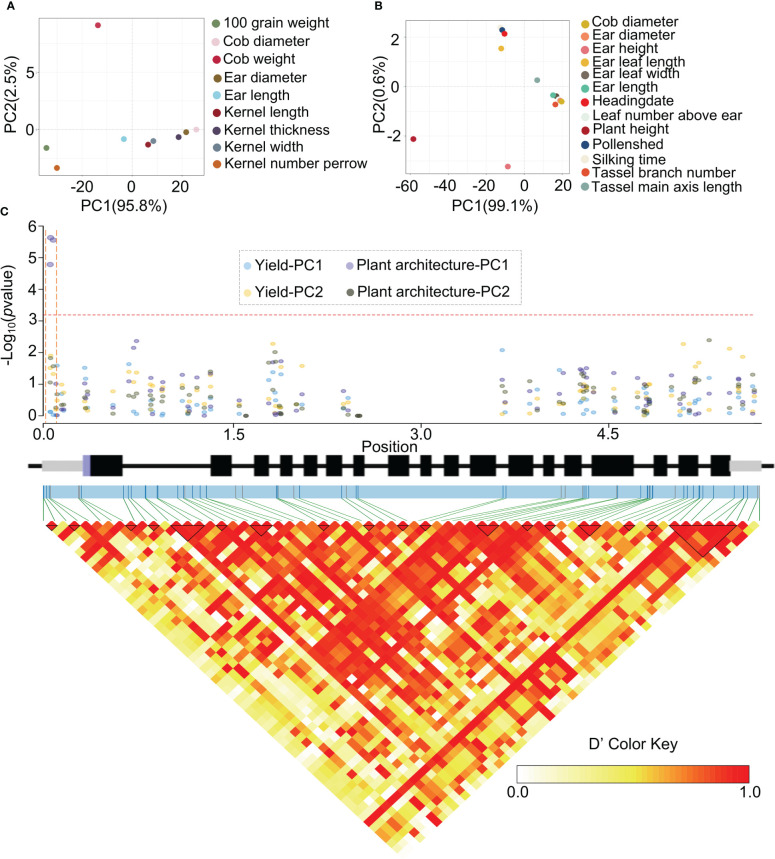
*ZmHOX32* association analysis. **(A)** PCA of maize yield. **(B)** PCA of plant architecture. **(C)** Association analysis of *Hox32* in four phenotypic data (Yield-pc1, Yield-pc2, Plant architecture-pc1, Plant architecture-pc2).

To clarify the functions of *ZmHOX32* in regulation of the agronomic traits of maize given the tight relationship between photosynthesis and agronomic traits, we performed the genome-wide association study (GWAS) with general linear model in a diverse association mapping panel of 690 maize inbred lines (Abe et al., 2012; Gui et al., 2020). Multiple agronomic traits of the maize association population had been investigated before. These traits could be classified into two categories with Yield and Plant architecture based on their effects on the maize phenotype. The Yield is composed of 9 traits that related to the kernel size and ear size, while the category Plant architecture contained the traits that associated to tassel branch number, leaf, and flowering time (**Figures 8A, B**). To reduce dimensionality of these complex traits, the Principal Component Analysis (PCA) were performed for the two main traits categories. The PC1 explained almost of the variation (95.8% and 99.1%) for categories Yield and Plant architecture, respectively (**Figures 8A, B**), which suggested that the Yield traits and Plant architecture were likely to be determined or represent by the PC1. The values of PC1 and PC2 of two categories all showed the normal distribution in the association population (shapiro.test, *P* = 1). Therefore, we used the PC1 and PC2 as phenotypic data, and performed a panel of genome-wide association studies (GWAS) by combining 4,069,278 SNPs. The four phenotypic data (Yield-PC1, Yield-PC2, Plant architecture-PC1, Plant architecture-PC2) identified 28, 824, 2671, 452 significant SNPs at the genome-wide level, respectively (**Supplementary Figures 3**, **4**). For *ZmHox32*, we found three SNPs located on the promoter region were significantly associated to the PC1 of Plant architecture (**Figure 8C**). The PC2 of two trait types all showed no association with the sequence variation in the population (**Figure 8C**), which may be associated to their low explained rate of variation (PCA) for the Yield and Plant architecture. The three SNPs that significantly associated to PC1 of Plant architecture could divide the population into three Haplotypes (Haps). The plants of Hap1 (CCC) showed significant higher plant height, higher ear height, larger ear leaf width, larger ear leaf length, larger tassel main axis length, more leaf number above ear, larger ear length, longer silking time, longer pollen shed time, and longer heading date than the plants of Hap3 (GTG) (**Supplementary Figure 5**). Tassel branch number, ear diameter and cob diameter showed no significant difference among Hap1, Hap2 (CTC) and Hap3 (**Supplementary Figure 5**). The Hap2 showed highest in most traits in category of Plant architecture, except in ear length, ear diameter and cob diameter. In summary, *ZmHOX32* likely to play an important role in regulation of plant architecture.

## Discussion

The HD-Zip III family exists in both C3 and C4 crops, plays an important role in regulating various cell differentiation processes in plants, including embryo morphogenesis, meristem formation, lateral organogenesis, lateral organ polarity establishment, and vascular system development, etc. (Prigge et al., 2005). Within this family, the regulatory mechanism of HOX32 influencing leaf morphogenesis has been studied in the C3 crops (rice and *Arabidopsis*) (Emery et al., 2003; Li et al., 2016). However, the function of HOX32 in C4 crop maize has not been studied. In present study, we focused on *ZmHOX32* in maize and found its high conservation in plants. As expected, *ZmHOX32* as one TF showed specific expression in nucleus, and constitutive expression in several tissues or organs, including the leaves at different growth stages or different leaf parts. Multiple *cis*-acting elements related to light-responsive were identified in promoter of *ZmHOX32*, suggesting a potential role in photosynthesis. The functions and the regulatory networks constructed for the interacting proteins and target genes of HOX32 also suggest a potential role in photosynthesis.

It has been studied that HOX32 is involved in the regulation of leaf morphogenesis and thus affects photosynthesis. In *Arabidopsis*, the homologous gene PHV was expressed in the abaxial side of cotyledons and center of the protovascular bundle (McConnell et al., 2001; Emery et al., 2003). Mutation of PHV caused changes in leaf polarity and the development of shoot and root apical meristems. Alteration in leaf morphology may indirectly affect photosynthesis efficiency. Overexpression of *OsHOX32* resulted in a variety of phenotype changes, including narrow adaxially curled leaves, reduced leaf angle, erect plant type, dwarf plants, and reduced chlorophyll levels, which all affected photosynthetic efficiency (Li et al., 2016). *OsHOX32* is the main target of microRNA166, the regulatory module may be involved in regulation of cell wall formation and vascular tissue development (Zhang et al., 2018b). The OsHOX32 protein could directly bind to the promoters of cinnamyl alcohol dehydrogenase (CAD) gene and cellulose synthase (CESA) gene, repressing their expression level and affecting leaf shape (Chen et al., 2021). In this study, to explore the regulatory pathway of *ZmHOX32* functioned on photosynthesis, tsCUT&Tag, an upgrade of ChIP-seq, was employed to investigate the downstream genes of *ZmHOX32*. The downstream genes of *ZmHOX32* were enriched in multiple gene families, such as WRKY, AUXIN, AP2/ERF, MYB, NAC, HB and VQ, which are related to leaf structure, signal transduction, and photosynthesis directly or indirectly. For examples, the inhibition of *OsSWNs* (encoding one NAC domain protein) expression in rice results in leaf drooping and reduced plant height (Yoshida et al., 2013). The mutation of *WUSCHEL-related homeobox1 (WOX1*) caused significant developmental defects in mid-lateral axis polarity and narrowed leaf width during leaf morphogenesis (Wang et al., 2020). In *Arabidopsis*, *AtTIFY4a* and *AtTIFY4b* regulate leaf development by affecting leaf size and leaf edge curvature (White, 2006). The drought tolerance of *JAZ7* can be induced by modulating photosynthesis, redox, amino acids, phytohormones, and defense metabolites in plants (Meng et al., 2019). We also focused on the genes upstream of *ZmHOX32*, and identified three transcription factors that function in the leaf. Previous studies showed that the transcription factor bHLH43 was related to the light regulation mechanism of carotenoids, and bHLH163 participated in the regulation of the tricarboxylic acid cycle by light (Eprintsev et al., 2022; Xiang et al., 2022). These potential effects on leaf development of the targets suggesting potential function of *ZmHOX32* in photosynthesis.

Protein-protein interaction study demonstrated that NAC, AP2/ERF, and MYB genes would interact with ZmHOX32 as co-factors. Current studies have found that the genes of the NAC family play important roles in different physiological developmental processes in plants, including stem tip meristem formation, leaf senescence, secondary cell wall formation, and hormone signaling (Grant et al., 2010; Yoshida et al., 2013; Ren et al., 2018; Zhang et al., 2018a). AP2/ERF family transcription factors regulate a variety of biological processes such as growth, development and differentiation, hormone signaling, and metabolic synthesis in different tissues (Feng et al., 2020). In the study of MYB family, it has been shown that *AtMYB76* regulates photosynthesis (Duan et al., 2017).

Here, we explored the upstream regulators, co-factors, and downstream targets of *ZmHOX32*, which for the first time construct the cascading regulatory network of *ZmHOX32* in maize. Based on such regulatory network, we uncovered functional roles of *ZmHOX32* in photosynthesis and plant architecture of maize.

## Data availability statement

The datasets presented in this study can be found in online repositories. The names of the repository/repositories and accession number(s) can be found below: BioProject: PRJNA909077 Accession: SRR22580376· SRR22580377· SRR22580378· SRR22580379.

## Author contributions

LL, XM, and WZ designed the project. XM, WZ, QJ, and ZS performed the experiments, data analysis, and collection of electronic resources. LL supported the work financially and participated in its planning. XM and WZ wrote the manuscript. All authors contributed to the article and approved the submitted version.

## Footnotes

1
http://bioinformatics.psb.ugent.be/webtools/plantcare/html/

2
http://www.ncbi.nlm.nih.gov/cdd/

3
http://systemsbiology.cau.edu.cn/agriGOv2/
