# Supplementary material for: ZmHOX32 is related to photosynthesis and likely functions in plant architecture of maize
Source: Front Plant Sci. 2023 Mar 22;14:1119678. doi: 10.3389/fpls.2023.1119678 (PMC10073575; doi:10.3389/fpls.2023.1119678)
Supplement: Supplementary file 1 [file DataSheet_1.pdf]

# *Supplementary Material*

**Supplementary Table 1.** Primers sequences information.

| | |
|---|---|
| ZmHOX32-F: | AGCAGATCTATCGATTCTAGAATGTCGACGGCGCTGG |
| ZmHOX32-R: | GCCCATGGCTCTAGAATCCACGAAGGACCAGTTCACG |

## Supplementary Figure

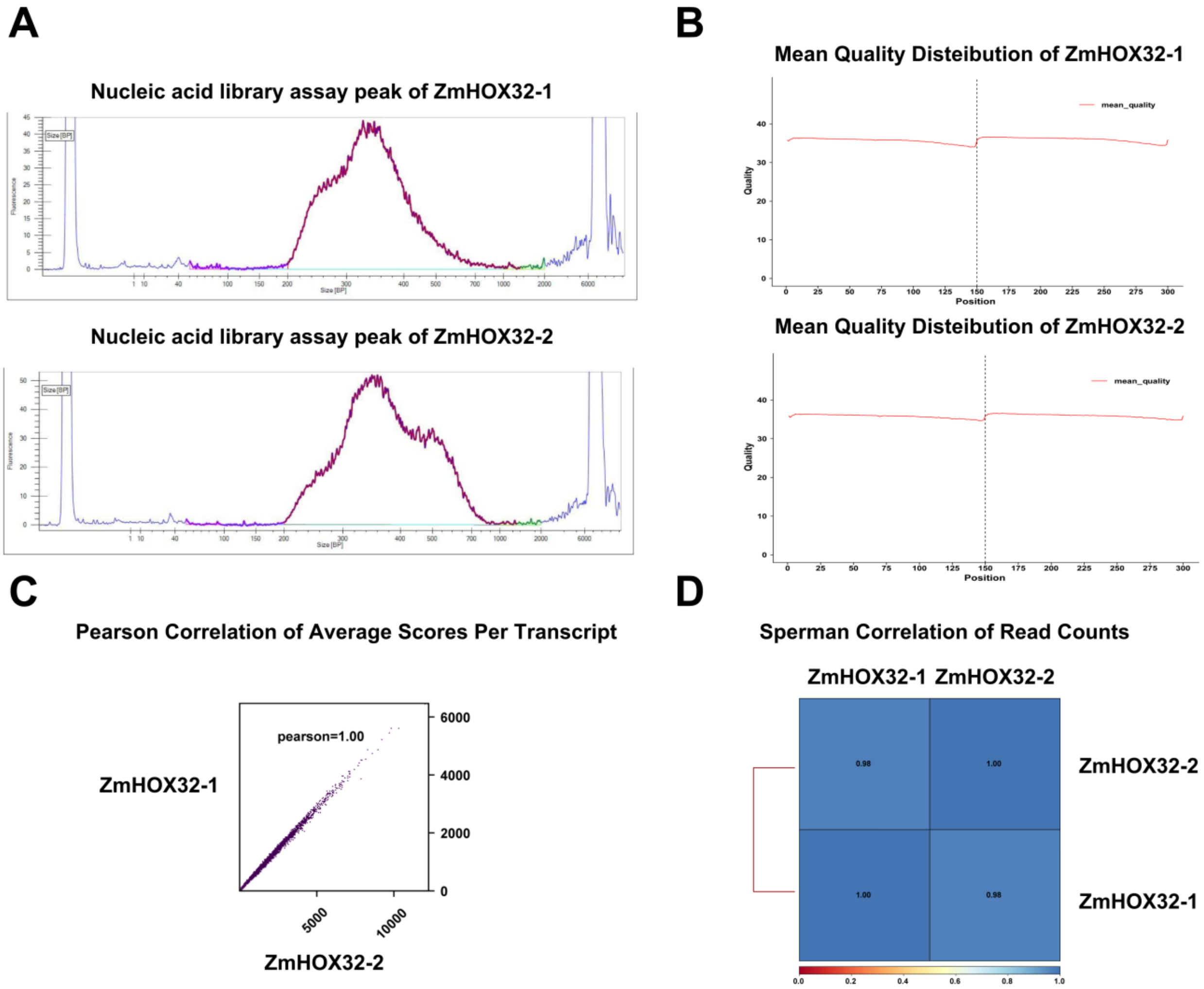


**Supplementary Figure 1. The l**ibrary and data quality of tsCUT&Tag of ZmHOX32. (A) Nucleic acid library assay peak map for ZmHOX32. (B) Library sequencing quality distribution of ZmHOX32. (C) and (D) Pearson Correlation Coefficient and Spearman Correlation Coefficient between two replicates.

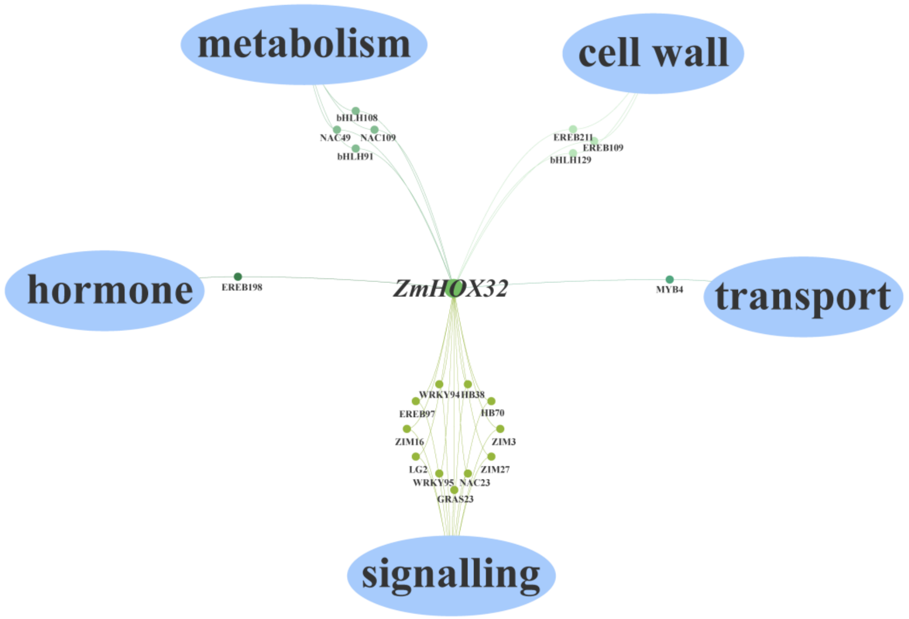


**Supplementary Figure 2.** Functional enrichment of *ZmHOX32* downstream target genes using the ChIP-seq data of 104 transcription factors.

A

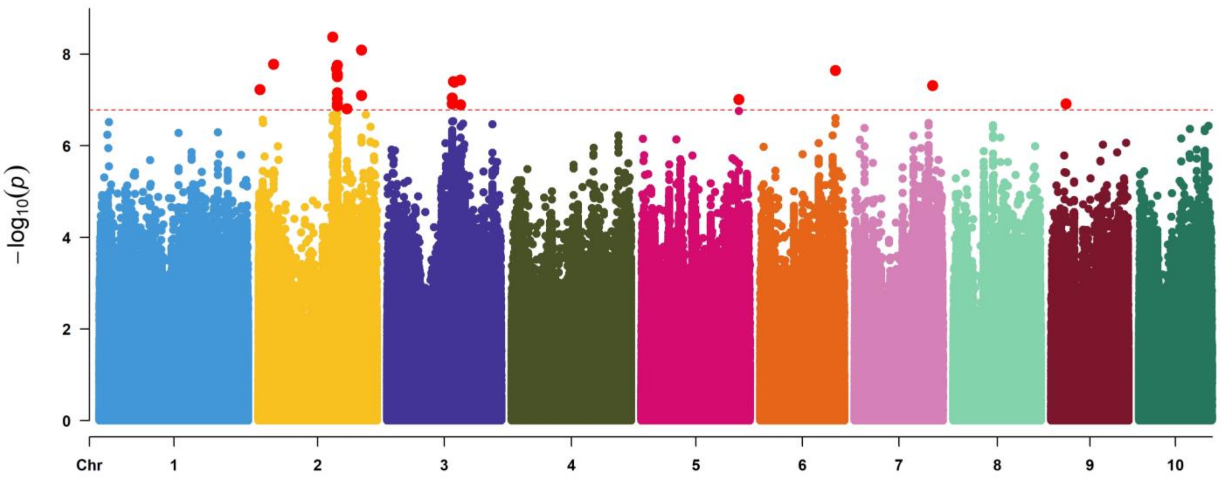


B

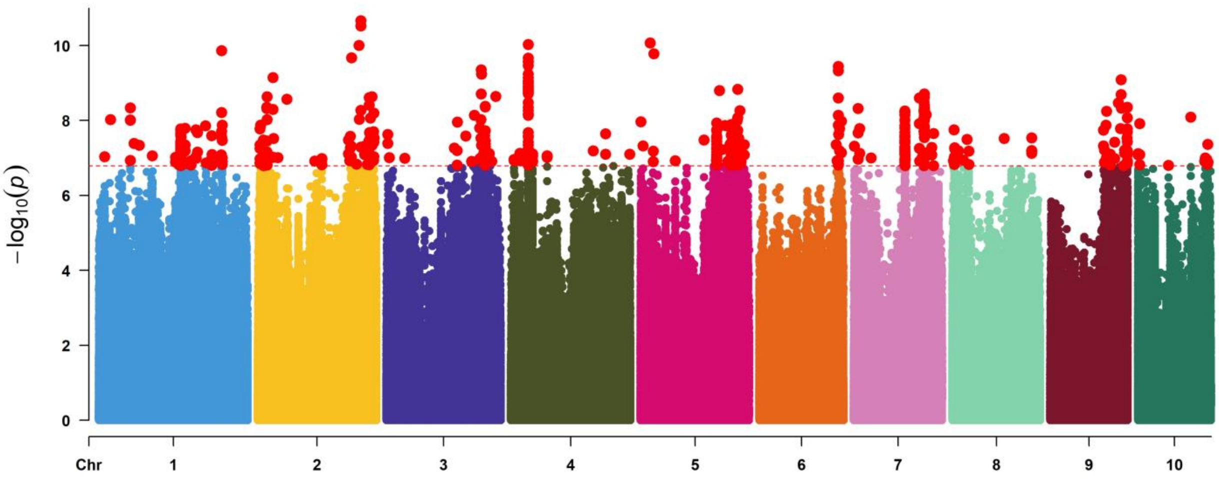


**Supplementary Figure 3.** Genome-wide association analysis of two phenotypic data. (A) Genome-wide association analysis of Yield-PC1. (B) Genome-wide association analysis of Yield-PC2.

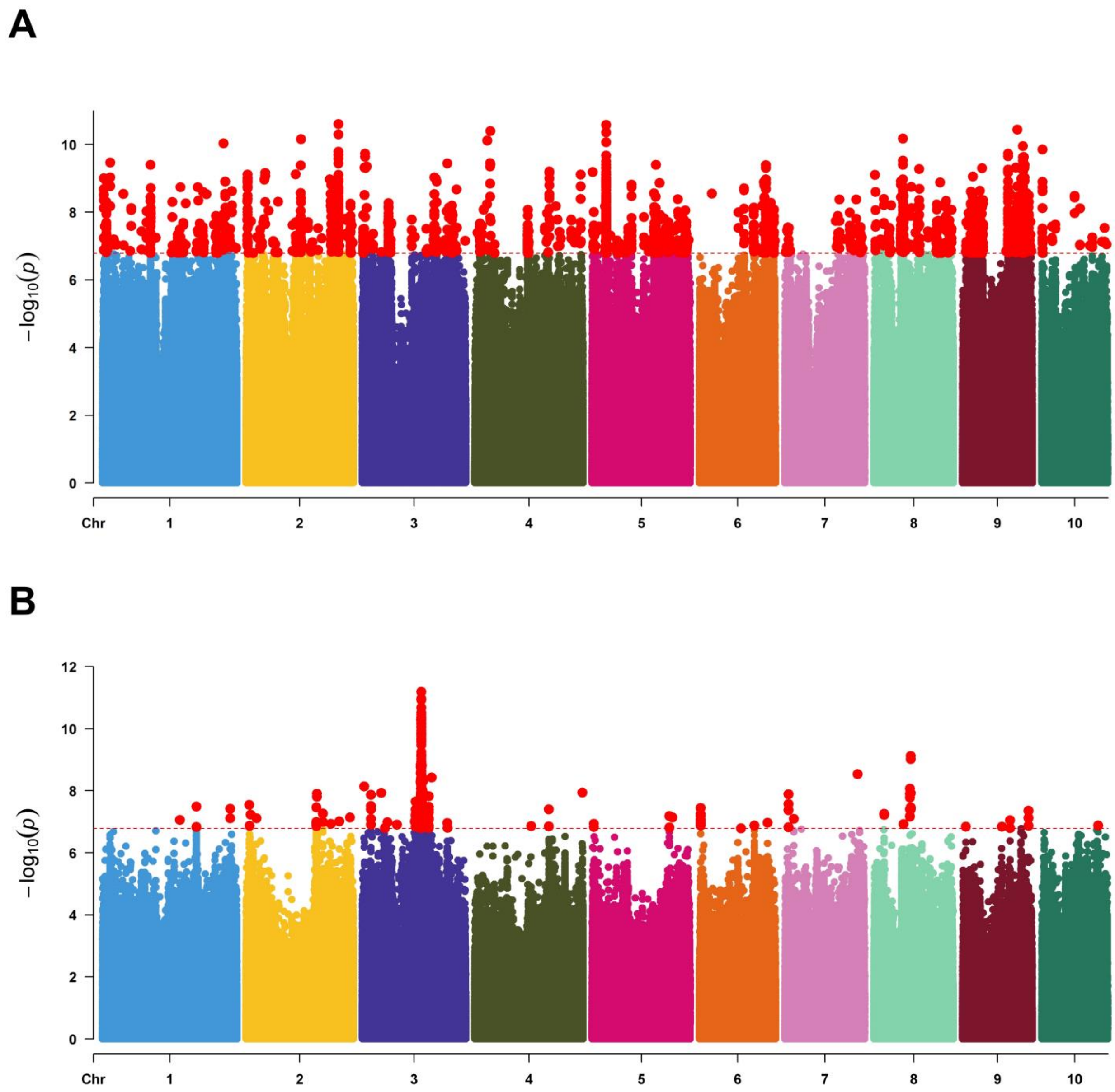


**Supplementary Figure 4.** Genome-wide association analysis of two phenotypic data. (A) Genome-wide association analysis of plant architecture-PC1. (B) Genome-wide association analysis of plant architecture-PC2.

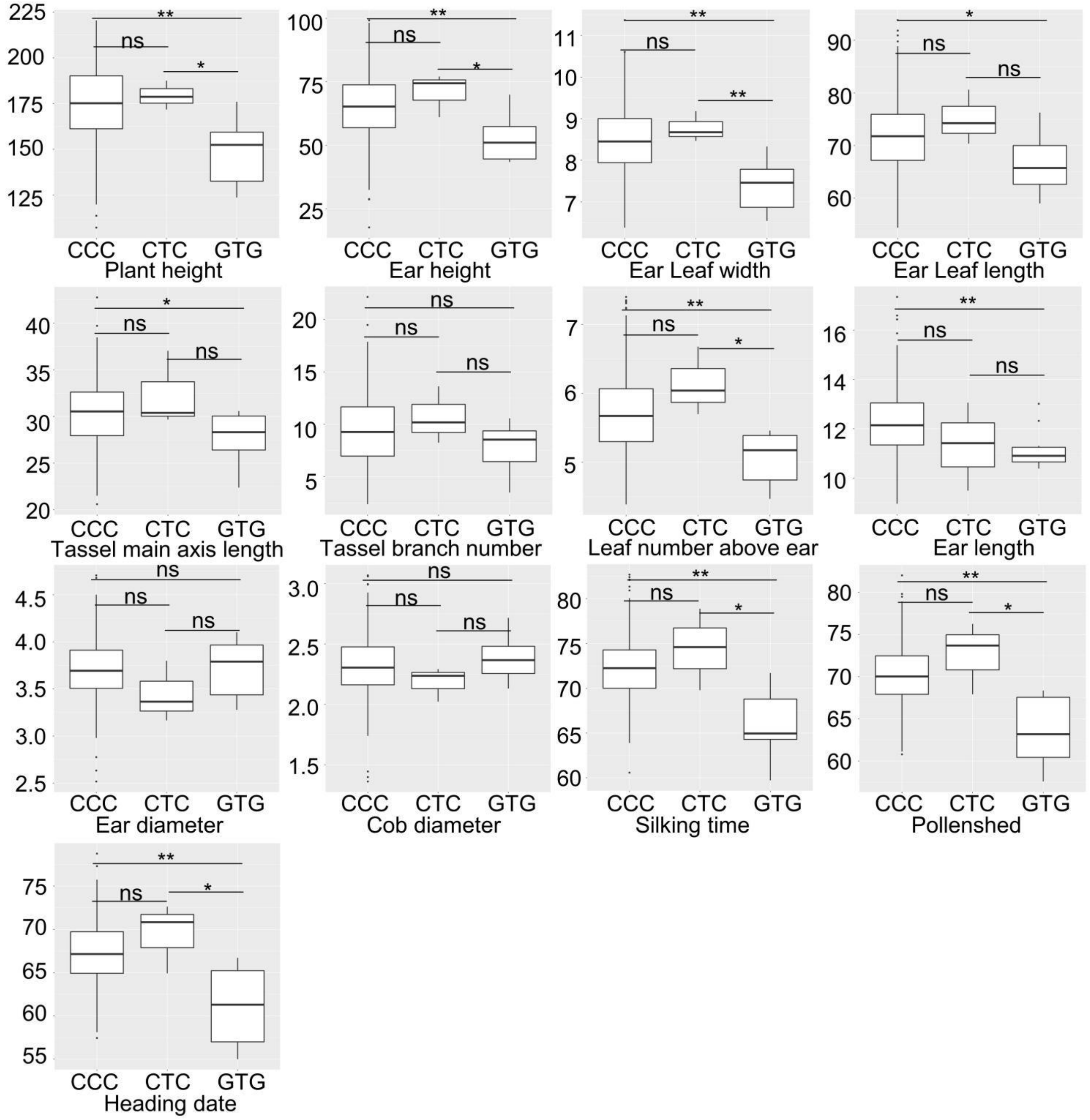


**Supplementary Figure 5.** The three SNPs of *ZmHOX32*, which are clearly associated with plant structural PC1, divide the population into three Haplotypes (Haps).
